# Study of Effect of Household Parental Smoking on Development of Acute Otitis Media in Children Under 12 Years

**DOI:** 10.5539/gjhs.v8n5p81

**Published:** 2015-08-31

**Authors:** Soroush Amani, Parastoo Yarmohammadi

**Affiliations:** 1 Department of Otolaryngology, Faculty of Medicine, Shahrekord University of Medical Sciences, Shahrekord, Iran; 2 Department of Health Education, Research Expert, Shahrekord University of Medical Sciences, Shahrekord, Iran

**Keywords:** acute otitis media, children, cigarette smoke

## Abstract

**Background and Aim:**

High prevalence of acute otitis media (AOM) in children represents a combination of the factors developing eustachian tube dysfunction and higher susceptibility to upper respiratory tract infections in children. This disease is relatively prevalent in Iran and much cost is spent annually to treat it. This study investigated the effect of household parental smoking on development of AOM in children under 12 years.

**Methods:**

In this case-control study all patients under the age of 12 years with AOM referring an ENT clinic in Shahrekord, southwest Iran between April 2014 and August 2014 were enrolled by convenience sampling. This study included two groups. Group 1 (G1) was exposed to parental smoking at home and group 2 (G2) was not. For the patients, a questionnaire of demographic data such as age and gender, the disease symptoms, parents’ education level, history of respiratory diseases, allergy, surgery (adenoidectomy, tonsillectomy, and tympanostomy), and household smoking was filled out by a specialist through interview.

**Results:**

In this study, 250 children 1-12 years with AOM, 145 in G1 and 105 in G2, were investigated. Clinical symptoms including fever (p=0.001) and hearing loss (p=0.014) were significantly more frequent in the children of G1 than G2, and otalgia, discharge, and tinnitus were similarly frequent in the two groups (p>0.05). Also, eardrum inflammation was more frequent in G1 than G2, with no significant difference (p>0.05). AOM was reported 70.3% in G1, which was higher than 26.7% reported in G2 (p=0.001). Also, asthma, recurrent ear pain, enlargement of the tonsils, and respiratory problems were more frequent in G1 than G2 (p<0.05).

**Conclusions:**

Parental smoking was a risk factor for AOM and respiratory problems and therefore the parents are recommended to avoid smoking near children to reduce the likelihood of AOM development and exacerbation in children.

## 1. Introduction

Otitis media (OM) is the second prevalent disease after viral upper respiratory tract infection in children and also the most common indication for antibiotic prescription (Marra, Patrick, Chong, & Bowie, 2006). Direct and indirect costs associated with OM management are high. In the USA, direct costs are estimated 3-5 billion dollars per year (Bondy, Berman, Glazner, & Lezotte, 2000). OM has two main types: suppurative or acute OM (AOM) and non suppurative or secretory OM or OM with effusion. These two main types of OM are interrelated. Acute infection is usually followed by residual inflammation and effusion predisposes children to recurrent infection. Middle-ear effusion is a characteristic of both AOM and secretory OM; in both conditions, an expression of the underlying middle-ear mucosal inflammation occurs. Recurrent OM, three or more episodes within six months, is associated with hearing deficits and speech delay. Factors thought to contribute to the occurrence of OM are age, male sex, race, genetic background, low socioeconomic status, lack of breastfeeding, exposure to cigarette smoke, older siblings, daycare, presence of respiratory allergy, and pneumococcal vaccination status (Engel, Straetemans, & Zielhuis, 2005). Birth weight, gestational age, and season of birth have also been investigated, but less consistent findings have been obtained (Kallio et al., 2007). Previous studies demonstrate that environmental tobacco smoke is an important indoor health risk, exposure to which may have harmful consequences such as asthma, atopic diseases, atherosclerosis, and OM (Jensen, Koch, Homoe, & Bjerregaard, 2013; Leatherdale, Smith, & Ahmed, 2008; Nuorti et al., 2000; Yilmaz, Caylan, & Karacan, 2012). Active and passive smoking increases the risk of certain respiratory tract infections and invasive diseases in adults (DiFranza, Aligne, & Weitzman, 2004). In children, passive smoking is associated with upper and lower respiratory tract infections, such as AOM, pneumonia, and bronchitis (Lanari et al., 2002). Several studies have shown that passive smoking is associated with increased prevalence of OM (Kum-Nji, Meloy, & Herrod, 2006). Passive smoking can increase bacteria adherence to the respiratory epithelium, depress local immune function, and decrease mucociliary action, and hence is potentially a risk factor of OM development (Arcavi & Benowitz, 2004). Harmful effects of passive inhalation of cigarette smoke on the incidence or severity of these problems have attracted researchers’ attention for many years. Since children’s most of the leisure time is spent at home, the constant exposure to cigarette smoke is considered as a predisposing factor for OM (Hawkins & Berkman, 2011). Despite the numerous reports on adverse effect of passive smoking, it has not been yet clarified whether smoke exposure increases the risk of OM in children. This information could contribute to motivating parents of OM prone children to stop smoking. Since this disease is relatively prevalent in Iran and stupendous costs are spent annually to treat it, we decided to investigate the effect of household parental smoking on development of AOM in children under 12 years.

## 2. Methods

### 2.1 Participants

In this case-control study all patients under the age of 12 years with AOM referring an ENT clinic in Shahrekord, southwest Iran between April 2014 and August 2014 were enrolled by convenience sampling. This study included two groups. Group 1 (G1) were exposed to parental smoking at home and group 2 (G2) were not.

The inclusion criteria were the incidence of AOM, no cerumen in the ear, and no old perforation of the tympanic membrane. The exclusion criteria consisted of anatomical problems underlying AOM, adenoid hypertrophy, and family history of weakness of the immune system (frequent infections other than AOM).

### 2.2 Materials

After the children were examined, a checklist was administered by interview to the parents of the children in the two groups. The questionnaire included items regarding the children’s data such as age, gender, the disease symptoms, parents’ education level, history of respiratory diseases, allergy, surgery (adenoidectomy, tonsillectomy, and tympanostomy) and parental smoking in terms of type and quantity.

Parental household smoking was determined by the existence of parents who currently smoked cigarettes in the household. Household exposure to smoking was coded as yes or no for the presence of smoking parents in the household, quantified by summing the number of cigarettes smoked per day.

### 2.3 Procedures

The research purposes were explained to all parents and informed consent was obtained from all of them. The children whose mothers accepted the conditions of the study entered the project. Ethical approval was given by Ethics Committee of Shahrekord University of Medical Sciences. The children were examined by an otolaryngologist and then he also interviewed the parents within 10 minutes to fill out a checklist.

### 2.4 Statistical Analysis

All analyses for this study were done using SPSS 17. Descriptive statistics were used for all variables. Chi-square test was used to evaluate the relationships. P-value of <0.05 (two tailed) was considered the level of significance.

## 3. Results

### 3.1 Children Demographics and Clinical Symptom

In this study, 250 children 1-12 years old with AOM, 145 in the G1 and 105 in the G2, were investigated. In the G1 and G2, respectively 57.2% and 62.9% were 1-6 years old and 42.7% and 37.1% were 6-12 years old. 39.3% and 50% of the G1 and G2, respectively, were girls. For clinical symptoms, in the G1, 92.4% were reported to have ear pain, 73.8% fever, and 29.7% hearing loss. In the G2, 93.3% were reported to have ear pain, 45.7% fever, and 16.2% hearing loss. By chi square, there was significant difference in fever and hearing loss between the two groups (p<0.05).

In the G1, 24.8% were reported to have mild redness, 68.3% severe inflammation, and 6.9% perforation of tympanic membrane. In the G2, 35.2% were reported to have mild redness, 52.4% severe inflammation, and 6.7% perforation of tympanic membrane. The affected ear in the two groups was mainly right ear. In both groups symptoms of cold were reported to present prior to starting AOM.

### 3.2 Recurrent AOM and Respiratory Problems During Sleep

70.3% of the G1 and 22.9% of the G2 had recurrent AOM with a significant difference between the two groups by chi square (p=0.001). 33.1% of the G1 and 26.7% of the G2 had history of allergy. 5.5% of the G1 and 1% of the G2 had history of asthma with a significant difference between the two groups (p=0.04). 70.3% of the G1 and 24.8% of the G2 had history of recurrent earache with a significant difference between the two groups (p=0.001). 62.8% of the G1 and 39% of the G2 had history of hypertrophy of tonsils with a significant difference between the two groups by chi square (p=0.001).

71.7% of the G1 and 42.9% of the G2 had respiratory problems during sleep with a significant difference between the two groups by chi square (p=0.001). The most commonly conducted surgeries in the two groups were tonsillectomy and adenoidectomy (Table 1).

**Table 1:** Distribution of individual and clinical variables between the two groups of children with otitis media in the two groups

Variables		Exposed to household parental smoking	Unexposed to household parental smoking	p-value
		No (%)	No (%)	
Age (yr)	6-Jan	83(57.2)	66(62.9)	**0.4**
	12-Jun	61(42.1)	39(37.1)	
Gender	Male	57(39.3)	52(49.5)	**0.09**
	Female	88 (60.7)	52(49.5)	
Clinical symptoms based on the pateints report	Ear pain	134 (92.4)	98(93.3)	**0.78**
	Fever	107 (73.8)	48(45.7)	0.001*
	Discharge	10 (6.9)	5(4.8)	**0.48**
	Hearing loss	43 (29.7)	17(16.2)	.014*
	Tinnitus	4 (2.8)	2(1.9)	**0.66**
Clinical symptoms based on the physician observation	Mild redness	36 (24.8)	37(35.2)	**0.1**
	Severe inflammation	99 (68.3)	55(52.4)	
	Eardrum rupture	10 (6.9)	7(6.7)	
	Moderate redness	0	1(1)	
Affected ear	Left	44(30.3)	27(25.7)	**0.8**
	Right	62(42.8)	46(43.8)	
	Both	39(26.9)	29(27.6)	
Cold symptoms prior to treatment initiation	Yes	119(82.1)	90(85.7)	**0.44**
	No	26(17.9)	15(14.3)	
Recurrent otitis	Yes	102(70.3)	24(22.9)	0.001*
	No	43(29.7)	81(77.1)	
Allergy history	Yes	48(33.1)	28(26.7)	**0.29**
	No	97(66.9)	76(72.4)	
Bronchitis history	Yes	1(0.7)	1(1)	**0.81**
	No	144(99.3)	104(99)	
Asthma history	Yes	8(5.5)	1(1)	0.49*
	No	137(94.5)	104(99)	
Recurrent ear pain history	Yes	102(70.3)	26(24.8)	0.001*
	No	43(29.7)	79(75.2)	
History of large tonsils or adenoids	Yes	91(62.8)	41(39)	0.001*
	No	54(37.2)	63(60)	
	Cleft palate surgery	0	1(1)	
Respiratory problems during sleep	Yes	104(71.7)	45(42.9)	0.001*
	No	41(28.3)	60(57.1)	

* p<0.05 significant;

^†^ Chi-square test.

### 3.3 Parental Demographics

Most fathers in the G1 had associate degree and BSc/BA and in the G2 high school certificate. The education level of mothers in the two groups was mainly high school certificate. The fathers’ and mothers’ job in the two groups was mainly self-employed and housewife, respectively (Table 2).

**Table 2:** Distribution of clinical variables and family characteristics between the two groups of children with acute otitis media in the two groups

Variables		Exposed to household parental smoking	Unexposed to household parental smoking	p-value
		No (%)	No (%)	
**Presence of allergic symptoms in nasal examination**	Yes	41(28.3)	21(20)	**0.13**
	No	104(71.7)	84(80)	
**Surgery history**	Adenoidectomy	1(0.7)	0	**0.49**
	Tonsillectomy	0	1(1)	
	Adenoidectomy and Tonsillectomy	17(11.7)	9(8.6)	
	None	126(86.9)	93(88.6)	
**Father’s education**	Primary	9(6.2)	7(6.7)	**0.79**
	Guidance	16(11)	10(9.5)	
	High school certificate	52(35.9)	45(42.9)	
	Associate and Bsc/BA	58(40)	35(33.3)	
	MSc/MA and PhD	10(6.9)	8(7.6)	
**Mother’s education**	Illiterate	1(0.7)	1(1)	**0.5**
	Primary	22(15.2)	10(9.5)	
	Guidance	21(14.5)	18(17.1)	
	High school certificate	61(42.1)	40(38.1)	
	Associate and Bsc/BA	39(26.9)	33(31.4)	
	MSc/MA and PhD	1(0.7)	3(2.9)	
**Father’s occupation**	Laborer	28(19.3)	19(18.1)	**0.13**
	Clerk	52(35.9)	31(29.5)	
	Unemployed	3(2.1)	4(3.8)	
	Self-employed	59(40.7)	40(38.1)	
	Teacher	2(1.4)	7(6.7)	
**Mother’s occupation**	Housewife	97(66.9)	77(73.3)	**0.48**
	Clerk	39(26.9)	22(21)	
	Teacher	9(6.2)	5(4.8)	
**Family’s economic status**	Good and acceptable	20(13.8)	22(21)	**0.16**
	Moderate	99(63.8)	70(66.7)	
	Poor and unacceptable	26(17.9)	12(11.4)	

For most children of the two groups, the family’s economic status was average and a significant association was seen between allergic symptoms and family’s good and acceptable economic status (p=0.001). In the G1, most parents smoked less than 10 cigarettes, and only 2.1% used other substances such as opium (Table 3).

**Table 3:** Distribution of smoking in families between the two groups of children with acute otitis media in the group exposed to household parental smoke

Variables		No (%)
**Number of used cigarettes**	Less than 10 cigarettes	**138(95.2)**
	Between 10 and 20 cigarettes	**7(4.8)**
**Other substances use**	Yes	**3(2.1)**
	No	**142(97.9)**
**Substance type**	Opium	**3(2.1)**
**Years of smoking**	Less than 10 years	**77(53.1)**
	Between 10 and 20 years	**63(43.4)**
	Between 20 and 30 years	**4(2.8)**

There was no significant association between the age, gender, affected ear, cold symptoms before AOM, history of allergy, history of bronchitis, allergic symptoms in nasal examinations, history of surgery, father’s education, mother’s education, father’s occupation, mother’s occupation, and family economic status in the two groups of children (p>0.05) (Tables 1-3).

## 4. Discussion

Recently there has been a renewed interest in the study of passive smoking on AOM. Since children are developing and exposure to pollutants could lead to adverse outcomes for them, therefore the present study investigated the effect of household parental smoking on development of AOM in children under 12 years.

In this study, the children with household parental smoking had more severe AOM and asthma, and more respiratory problems during sleep. In a study, the children with reportedly more frequency of asthma, had a smoker family member (Hawkins & Berkman, 2011). Smoking by each family member is thought to considerably increase the risk of acquiring OM in children (Jones, Hassanien, Cook, Britton, & Leonardi-Bee, 2012). In another study, the children of 3-11 years exposed to passive cigarette smoke in the USA repeatedly acquired ear infection (three times and more a year) (Haggard, Gannon, & Birkin 2002). In a similar study on 70 patients referring an ENT clinic, 49 patients had exposure to cigarette smoke due to parents’ smoking. Out of 49 patients with OM, the majority were under eight years, which could be explained by the children of younger ages being present near and dependent on their parents and hence increased continuous exposure to cigarette smoke. Also, the prevalence of AOM has been reported significantly higher in the children with smoking parents (Einolghozati, Adami Dehkordi, & Sharifi Dalloee, 2007; Teele, Klein, & Rosner, 1980). In another study, out of 114 enrolled children of 3–8 years with OM, 74% had some exposure to cigarette smoke by their urinary cotinine level. In a large study on 3833 school and preschool children of 2-11 years old, the prevalence of the secretory OM was reported respectively 9.1% and 14.1% (Naini, Naini, & Vazirnezam, 2002). A study indicated that 34.4% of the children with AOM and 19.3% of the children in control group had family history of smoking. Smoking frequency was significantly lower in healthy children’s families than the families of children with AOM (Berjis, Abdeyazdan, Okhovat, Gholami Ghasri, & Salem, 2011). Inconsistent with the present study, a systematic study’s findings indicated that parents’ smoking did not contribute to increasing AOM prevalence (Cook & Strachan, 1999).

In the USA, the OM prevalence was reported 86.1% in children over 13 years exposed to household smoking in 2006. This study argued that the growing trend of OM can be reduced with the increase in smoking-free homes (Alpert, Behm, Connolly, & Kabir, 2011). Also, different studies have demonstrated that, the children are more exposed to cigarette smoke at home and hence the interventions aimed at prohibiting smoking at home could reduce children’s exposure to cigarette smoke (Greenberg et al., 1994; Matt et al., 2004). In a study, the children, referring an ENT clinic in Hungary more frequently, had one parent and/or both parents smoking and were at higher risk of OM recurrence. Also, this study found that maternal employment (full-time vs. part-time) status increased the risk of recurrent OM by nearly four times (Csakanyi, Czinner, Spangler, Rogers, & Katona, 2012). In our study there was no significant association between OM severity and parents’ socioeconomic status and education. A study on 5762 children of 10 to 13 years old in California, the USA showed that exposure to environmental smoking, alongside other risk factors, increased the likelihood of wheezing attacks. In this study, cigarette smoke exposure and family history of asthma and allergies were associated with asthma development in children (Gilliland, Li, & Peters, 2001). In a study conducted in New York, the USA, the girls developed respiratory complications more frequently than boys and the complications were more frequent in the children with illiterate parents (Skolnick, Vomvolakis, Buck, Mannino, & Sun, 1998), which is not consistent with our findings. We found a significant association only between the presence of allergic symptoms and acceptable economic status, and no significant association was seen between AOM severity and economic status and parents’ education.

A study in the UK indicates that the OM incidence in children has declined in recent years, which could represent the decreased number of smoking parents. However, smoking parents are still reported as the main source of concern for children’s health in the UK. One of the potential limitations of our study was that the exposure to cigarette smoke was measured through interview. But, in addition to self report, urinary cotinine levels could be used to measure the exposure to cigarette smoke. Also, parents’ self report could not be sufficiently reliable because they are likely to be unwilling to talk of smoking.

In the present study the patients were enrolled from a wide age range (1-12 years), although a study with larger sample size could definitely yield more reliable findings. Moreover, we adopted a convenience sampling within a short period of time and a random sampling from a larger study population is clearly more valuable from research perspectives. In this study, in addition to study of cigarette smoke effect on AOM development, other potentially influencing factors including family’s economic status were measured, as well. As the present study demonstrated, cigarette smoking is a risk factor of AOM and exacerbation of its symptoms. Therefore, prospective epidemiologic studies with large sample size are required to *inclusively* investigate adverse effects of cigarette smoking so that appropriate healthy behaviours could be disseminated in community.

## 5. Conclusion

The present study indicated that, children’s exposure to household parental smoking is a risk factor of AOM and respiratory problems. Therefore, primary prevention by decreasing risk factors including exposure to cigarette smoke is the key to declining OM’s burden in childhood. In this regard, more efforts should be made to promote cigarette smoke-free environment to decrease OM and respiratory diseases in children.
